# Retinoid X receptor gamma (RXRG) is an independent prognostic biomarker in ER-positive invasive breast cancer

**DOI:** 10.1038/s41416-019-0589-0

**Published:** 2019-09-27

**Authors:** Chitra Joseph, Sara Al-Izzi, Mansour Alsaleem, Sasagu Kurozumi, Michael S Toss, Maariya Arshad, Fang Qin Goh, Ibraheem M. Alshankyty, Mohammed A. Aleskandarany, Simak Ali, Ian O. Ellis, Nigel P. Mongan, Andrew R. Green, Emad A. Rakha

**Affiliations:** 1 Nottingham Breast Cancer Research Centre, Division of Cancer and Stem Cells, School of Medicine, University of Nottingham and Nottingham University Hospital NHS Trust, Nottingham, UK; 2 Histopathology Department, Faculty of Medicine, Menoufia University, Shebin El Kom, Egypt; 3 Faculty of Applied Medical Sciences, King Abdulaziz University, Jeddah, Saudi Arabia; 4 Faculty of Medicine, Department of Surgery & Cancer, Imperial College London, London, UK; 5 Cancer Biology and Translational Research, Faculty of Medicine and Health Sciences, University of Nottingham, Nottingham, UK; 6 Department of Pharmacology, Weill Cornell Medicine, New York, NY 10065 USA

**Keywords:** Breast cancer, Prognostic markers

## Abstract

**Background:**

Retinoid X Receptor Gamma (RXRG) is a member of the nuclear receptor superfamily and plays a role in tumour suppression. This study aims to explore the prognostic significance of RXRG in breast cancer.

**Methods:**

Primary breast cancer tissue microarrays (*n* = 923) were immuno-stained for RXRG protein and correlated with clinicopathological features, and patient outcome.

**Results:**

Nuclear RXRG expression was significantly associated with smaller tumour size (*p* = 0.036), lower grade *(p* < 0.001), lobular histology (*p* = 0.016), lower Nottingham Prognostic Index (*p* = 0.04) and longer breast cancer-specific survival (*p* < 0.001), and longer time to distant metastasis (*p* = 0.002). RXRG expression showed positive association with oestrogen receptor (ER)-related biomarkers: GATA3, FOXA1, STAT3 and MED7 (all *p* < 0.001) and a negative correlation with the Ki67 proliferation marker. Multivariate analysis demonstrated RXRG protein as an independent predictor of longer breast cancer-specific survival and distant metastasis-free survival. In the external validation cohorts, *RXRG* expression was associated with improved patients’ outcome (*p* = 0.025). In ER-positive tumours, high expression of RXRG was associated with better patient outcome regardless of adjuvant systemic therapy. ER signalling pathway was the top predicted master regulator of RXRG protein expression (*p* = 0.005).

**Conclusion:**

This study provides evidence for the prognostic value of RXRG in breast cancer particularly the ER-positive tumours.

## Introduction

Breast cancer is the most common cancer among women worldwide.^1^ Oestrogen receptor (ER) and progesterone receptor (PR), which are members of the nuclear receptor superfamily of transcription factors, are important in predicting prognosis and establishing therapeutic strategies for breast cancer treatment. Recent studies have revealed growing evidence of the involvement of nuclear receptors, other than ER and PR, in breast cancer development and progression.^2,3^ Drugs targeting nuclear receptors are widely used in the clinic for treating patients.^4^ Expression levels of some nuclear receptors, such as thyroid hormone receptor beta (THRb), COUP transcription factor 2 (COUP-TF2), peroxisome proliferator-activated receptor gamma (PPARG) and liver receptor homologue-1 (LRH-1), are associated with clinicopathological variables and can predict outcome in tamoxifen-treated patients.^5^ The glucocorticoid receptor (GR) in breast cancer exerts anti-proliferative and anti-apoptotic activities and its overexpression is associated with features characteristic of longer survival.^6,7^ Moreover, in tamoxifen-treated ER-positive breast cancer, androgen receptor (AR; also a member of the nuclear receptor superfamily) status has prognostic value and it is reported to be a crucial factor in deciding treatment regime.^8^ With these important roles in breast cancer, other nuclear receptors could therefore provide additional therapeutic targets for breast cancer management.^9–11^

Retinoids derived from vitamin A are signalling molecules that play important roles in cell differentiation and proliferation^12^ and act via retinoic acid receptors (RARs) and retinoid X receptors (RXRs), which are members of the nuclear receptors superfamily. Retinoids are well documented for their ability to induce differentiation and arrest proliferation in cancer.^12,13^ The RXR family are known to form heterodimers with other nuclear receptors, including the vitamin D receptor (VDR), peroxisome proliferator-activated receptors (PPARs) and RARs.^11^ There are three subtypes of the Retinoid X Receptor (RXR), namely RXR Alpha (RXRα; NR2B1), RXRβ (NR2B2) and RXRγ (NR2B3).^14^ These receptors have tumour suppressor properties, particularly as their ligand 9*-cis*-retinoic acid,^12^ and impede cellular proliferation.^15^ Moreover, the RXR family are involved in mediating the anti-proliferative effects of retinoic acid (RA) as partners of RARB and RARA.^12^

RXRG has been demonstrated to modulate cellular differentiation and apoptosis in different tumour types. For example, enhanced expression of RXRG was associated with increased apoptosis in ovarian cancer.^16^ Epigenetic silencing of RXRG correlated with decreased overall survival in lung cancer.^17^ In ovarian cancer tumour models, RXRG activation re-sensitises ovarian carcinoma cells to apoptosis. However, the mechanism by which this occurs is still unclear. With minimal toxicity both in vitro and in vivo, novel RXR family members (rexinoids), have been reported to suppress breast cancer development in several animal models and have been extensively evaluated either alone or in combination with selective ER modulators.^18^ One RXRG partner, RARA was shown to influence the ERα transcriptional complex in oestrogen-treated MCF-7 breast cancer cells.^19,20^ Altogether, these findings indicate that RXRG could have a function in tumour pathogenesis and could potentially be promising cancer therapeutics.

Therefore, this study aimed to investigate the potential prognostic role of RXRG in breast cancer with a focus on the luminal ER-positive class.

## Methods

### Study cohort

This study was conducted on a large cohort (*n* = 923) of primary breast cancer from patients who presented to Nottingham City Hospital with available clinicopathological data, as previously described.^21–23^ Treatment and outcome data, including breast cancer-specific survival and distant metastasis-free interval was maintained on a prospective basis. Breast cancer-specific survival was defined as the duration (in months) from the date of primary surgery to the time of death because of breast cancer. Distant metastasis-free interval was defined as the duration (in months) from primary surgical treatment to the occurrence of first distant recurrence.

### Evaluation of RXRG protein expression

**Fig. 1:**
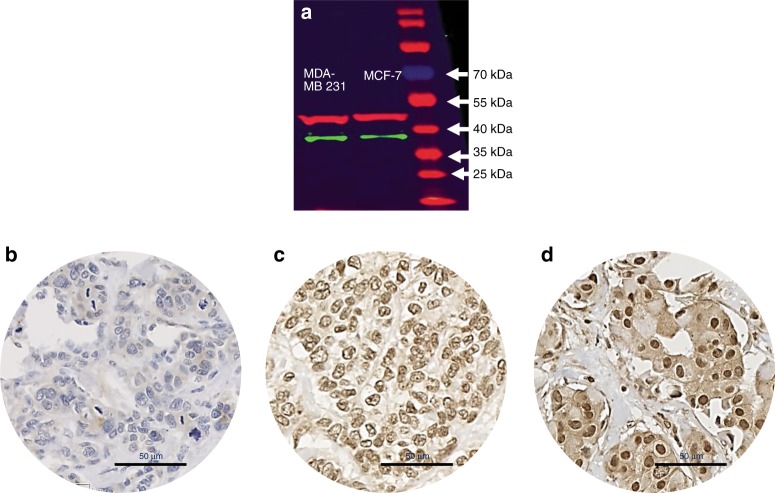
Western blot and immunohistochemical expression of RXRG in breast cancer. **a** Western blotting results for RXRG expression in MCF-7 and MDA-MB231 breast cancer cell lines using rabbit polyclonal antibody (Abcam, ab15518). Green and red bands represent RXRG and the house-keeping Beta-Actin, respectively. RXRG protein expression in breast cancer tissue microarrays cores. **b** Negative/no staining **c** showing low expression and **d** showing high immunoreactivity. Images are at x40 magnification

RXRG protein expression was evaluated using immunohistochemistry (IHC) preceded by validation of the rabbit RXRG antibody (Abcam, ab15518) specificity using western blot. For the latter, cell lysates of MDA-MB-231 and MCF-7 cell lines (obtained from the American Type Culture Collection; Rockville, MD, USA) were incubated with the primary antibody at 1:700 dilution. The specificity of the antibody was validated with a single specific band at the predicted molecular weight (39 kDa, Fig. 1a).

For evaluation of the morphological pattern of protein expression and suitability of tissue microarrays for its assay, immunohistochemistry was assessed in full-face breast cancer tissue sections (*n* = 10). Tumour samples were arrayed onto tissue microarrays as previously described.^21^ Four micrometre sections from the tissue microarrays and full-face sections were immunohistochemically stained using the Novolink Max Polymer Detection system (Leica, Newcastle, UK). The antibody was incubated 24 h at the concentration of 1:300.

The modified Histo-score (*H-*score) method was used in assessing immunohistochemistry staining, taking the staining intensity and percentage positivity into account.^24^ High-resolution digital images were generated via scanning the stained slides using Nanozoomer (Hamamatsu Photonics, Welwyn Garden City, UK) at x20 magnification to facilitate the scoring of the tissue microarrays cores. The sections were blindly double scored by two researchers, including a consultant histopathologist for ~25% cores to assess inter-observer concordance. Inter-observer agreement was determined, and the intra-class correlation co-efficient was 0.83, indicating an excellent concordance between scorers. Moreover, the discordant cases were re-scored by the both observers and a consensus score was agreed and assigned. Biomarkers closely relevant to breast cancer carcinogenesis, progression and outcome were also available for this cohort of patients (See Tables 2 and 3). Immunohistochemistry staining and dichotomisation of these biomarkers were used as per previous publications.^6,22,25–33^

### Gene expression cohorts

The clinicopathological significance of *RXRG* mRNA expression was assessed using a subset (*n* = 150) of the Nottingham series that was included in the Molecular Taxonomy of Breast Cancer International Consortium (METABRIC) data set.^34^ The aim of this investigation is to understand the molecular biology of RXRG protein expression as an end product, therefore, the analysis was completed utilising cases with RXRG protein expression. The definition of cases into low versus high groups was based on RXRG protein expression.

External validation was performed using the Breast Cancer Gene-Expression Miner v4.0 (bc-GenExMiner v4.0),^35^ as previously described.^33,36^ Breast cancer cases data set (*n* = 818) within The Cancer Genome Atlas (TCGA)^37^ was also used for external validation of *RXRG* mRNA expression. Patient outcome following systemic treatment was further validated using KM Plotter (*n* = 3951).^38^

### Pathway analysis

Differential gene expression between *RXRG* negative and positive cases was assessed using the Robina implementation of EdgeR.^39^ Differential expression with >2-fold difference and a false discovery rate of *q* < 0.05 between *RXRG*-negative and -positive cases were considered significant. Webgestalt (http://www.webgestalt.org) was used to annotate the differential gene expression list and to identify over-represented gene ontologies and pathways.^40^

### Statistical analysis

IBM SPSS 22.0 (Chicago, IL, USA) software was used for statistical analysis. The *H*-scores of expressions of nuclear RXRG did not follow a normal distribution. For this reason, expression of RXRG protein was used to define patient groups based on prediction of breast cancer-specific survival using X-tile (http://tissuearray.org; Yale University, USA).^41^ Chi-squared test was used to evaluate the association between expression of other biomarkers and the clinicopathological parameters. Correlation between RXRG and ER/PR expression was analysed using Spearman’s correlation co-efficient test. Association between RXRG expression, clinicopathological parameters and other related biomarkers using the continuous *H*-score were evaluated.

Kaplan–Meier analysis with log-rank test for significance was performed to assess breast cancer-specific survival and distant metastasis-free interval. Interaction between RXRG and ER was evaluated using Cox regression model, which was also used for multivariate survival analysis with adjustment of covariates to test independence from standard prognostic factors in breast cancer (nodal stage, tumour grade, tumour size, ER level of expression (defined as percentage of positive tumour cells), and Ki67. The STRING database (http://string-db.org)^42^ was used to evaluate the interaction with RXRG and other nuclear receptors in steroid signalling pathways. The *p*-values were adjusted using Bonferroni correction for multiple testing. A *p-*value of <0.05 was considered significant.

This study obtained ethics approval by the North West–Greater Manchester Central Research Ethics Committee under the title: Nottingham Health Science Biobank (NHSB), reference number 15/NW/0685. All samples from Nottingham used in this study were pseudo-anonymised and collected prior to 2006 and stored in compliance with the UK Human Tissue Act.

## Results

### RXRG protein expression

Full-face tissue sections (Supplementary Fig. 1a–c) showed high RXRG expression in the normal glandular epithelium (Supplementary Fig. 1b). In contrast, low RXRG immunopositivity was observed in the nuclei of invasive cancer cells (Supplementary Fig. 1c), with some malignant cells additionally featuring cytoplasmic staining. On tissue microarrays, RXRG protein expression levels varied from absent to high (Fig. 1b–d). In the 923 scorable cores, the cutoff points of the RXRG nuclear *H*-score was set at 175 by X-tile analysis, where low expression is defined as *H*-scores <175 and high expression as *H*-scores ≥175. Low RXRG nuclear expression was observed in 458/923 (49.6%) cases and high expression was observed in 465/923 (50.4%) cases. Low *RXRG* mRNA expression was found in 73/150 (49%), whereas high *RXRG* mRNA expression was observed in 77/150 (51%) cases.

### Relationship between RXRG protein expression and clinicopathological variables

In the whole cohort and ER-positive sub-cohort, RXRG was associated with features of favourable prognosis, including smaller tumour size (*p* = 0.036), lower histological grade (*p* < 0.00001), less pleomorphism (*p* = 0.042), lower mitotic scores (*p* < 0.00001), lobular and special tumour types of excellent prognosis (*p* = 0.016), and lower Nottingham Prognostic Index (*p* < 0.05; Table 1). Moreover, significant association was observed with breast cancer molecular intrinsic subtypes (*p* < 0.00001 and *p* = 0.009), for the whole series and ER-positive tumours, respectively (Table 1). High RXRG expression was primarily observed in luminal A tumours (136/214, 63.6%), while it was less expressed in HER2+ and triple-negative breast cancer.

**Table 1:** Associations between RXRG expression and clinicopathological features in the whole series, ER-positive and ER-Negative breast cancer series

Parameters	RXRG expression whole cohort			RXRG expression ER-positive cohort			RXRG expression ER-negative cohort		
	Negative/ low expression *N* (%)	High expression *N* (%)	*p-*value (*χ*^2^)	Negative/ low expression *N* (%)	High expression *N* (%)	*p*-value (*χ*^2^)	Negative/ low expression *N* (%)	High expression *N* (%)	*p-*value (*χ*^2^)
Age at diagnosis (years)									
<50	167 (51.2)	159 (48.8)	1.473 (0.520)	94 (43.3)	123 (56.7)	1.239 (0.682)	72 (68.6)	33 (31.4)	0.123 (2.673)
≥50	291 (48.7)	306 (51.3)		225 (46.7)	257 (53.3)		66 (57.9)	48 (42.1)	
Histological grade									
1	52 (35.6)	94 (64.4)	**<0.00001** (44.423)	49 (35.8)	88 (64.2)	**<0.00001** (25.929)	2 (40.0)	3 (60.0)	0.530 (1.271)
2	130 (40.8)	189 (59.2)		122 (39.9)	184 (60.1)		8 (61.5)	5 (38.5)	
3	273 (60.9)	175 (39.1)		145 (58.5)	103 (41.5)		128 (63.6)	71 (35.7)	
Tubules									
1	11 (26.2)	31 (73.8)	**0.004** (13.895)	11(27.5)	29 (72.5)	0.172 (6.284)	0 (0.0)	1 (100.0)	0.376 (1.959)
2	140 (46.1)	164 (53.9)		123 (44.2)	155 (55.8)		17 (68.0)	8 (32.0)	
3	289 (53.4)	252 (46.6)		169 (48.0)	183 (52.0)		120 (63.5)	69 (36.5)	
Pleomorphism									
1	5 (23.8)	16 (76.2)	**<0.00001** (23.960)	5 (26.3)	14 (73.7)	**0.042** (10.294)	0 (0.0)	1 (100.0)	0.406 (1.803)
2	144 (41.4)	204 (58.6)		136 (40.6)	199 (59.4)		8 (66.7)	4 (33.3)	
3	291 (56.2)	227 (43.8)		162 (51.3)	154 (48.7)		129 (63.9)	73 (36.1)	
Mitosis									
1	111 (36.0)	197 (64.0)	**<0.00001** (53.653)	107 (36.0)	190 (64.0)	**<0.00001** (22.597)	4 (44.4)	5 (55.6)	0.170 (3.452)
2	77 (43.3)	101 (56.7)		67 (42.4)	91 (57.6)		10 (50.0)	10 (50.0)	
3	252 (62.8)	149 (37.2)		129 (60.0)	86 (40.0)		123 (66.1)	63 (33.9)	
Stage									
I	280 (50.5)	275 (49.5)	1.69 (0.337)	203 (47.6)	221 (52.4)	1.064 (2.200)	80 (60.6)	52 (39.4)	0.522 (1.300)
II	141 (49.1)	146 (50.9)		97 (43.7)	125 (56.3)		43 (68.3)	20 (31.7)	
III	34 (47.2)	38 (52.8)		19 (38.0)	31 (62.0)		15 (68.2)	7 (31.8)	
Tumour size									
<2.0 cm	182 (42.8)	243 (57.2)	**0.0005** (15.355)	143 (40.6)	209 (59.4)	**0.036** (7.550)	38 (54.3)	32 (45.7)	0.071 (3.609)
≥2.0 cm	274 (55.8)	217 (44.2)		174 (51.0)	167 (49.0)		100 (67.6)	48 (32.4)	
Histological type									
Ductal	403 (53.3)	353 (46.7)	**0.0001** (29.455)	277 (49.5)	283 (50.5)	**0.016** (19.281)	125 (64.8)	68 (35.2)	0.071 (10.161)
Lobular	32 (32.3)	67 (67.7)		32 (33.0)	65 (67.0)		0 (0.00)	2 (100.0)	
Medullary-like	12 (57.1)	9 (42.9)		1 (50.0)	1 (50.0)		11 (57.9)	8 (42.1)	
Special type^a^	8 (22.2)	28 (77.8)		6 (18.8)	26 (81.3)		2 (100.0)	0 (0.0)	
IHC subtypes									
ER+/HER2– low proliferation	78 (36.4)	136 (63.6)	**<0.00001** (37.474)	78 (36.4)	136 (63.6)	**0.009** (14.564)			0.103 (2.849)
ER+/HER2 – high proliferation	147 (50.3)	145 (49.7)		147 (50.3)	145 (49.7)				
Triple negative	102 (68.0)	48 (32.0)					102 (68.0)	48 (32.0)	
HER2 +	71 (57.3)	53 (42.7)					31 (55.4)	25 (44.6)	
Nottingham Prognostic Index									
GPG	105 (39.2)	163 (60.8)	**0.0004** (19.294)	101 (39.8)	153 (60.2)	**0.040** (6.538)	3 (30.0)	7 (70.0)	0.051 (5.943)
MPG	260 (52.5)	235 (47.5)		165 (48.1)	178 (51.9)		95 (62.9)	56 (37.1)	
PPG	91 (59.5)	62 (40.5)		51 (45.7)	45 (46.9)		40 (70.2)	17 (29.8)	

Significant *p-*values are highlighted in bold;

*GPG* good prognostic group, *MPG* moderate prognostic group, *PPG* poor prognostic group

^a^Special types of excellent prognosis (invasive tubular, invasive cribriform, invasive mucinous, invasive papillary carcinoma)

There was a significant positive linear correlation between RXRG and ER expression in the whole cohort and in ER-positive tumours (*r* = 0.30, *p* < 0.0001 and *r* = 0.20, *p* = 0.016, respectively). Similar results were observed with PR expression (*r* = 0.20, *p* = 0.014 and *r* = 0.17, *p* = 0.016; respectively). High-nuclear RXRG expression showed significant positive association with ER and PR positivity (*p* < 0.0001 and *p* = 0.018, respectively), while negative association was observed with basal cytokeratin CK5/6 (*p* = 0.020; Table 2). High expression of RXRG was positively associated with luminal subtype-related biomarkers in both the whole cohort and ER-positive tumours, including ER-chromatin interaction regulator Forkhead box protein A1 (FOXA1; *p* < 0.00001) and human brain-expressed X-linked1 (BEX1; *p* < 0.00001). Significant positive associations were observed with cell cycle regulatory proteins such as GATA3 (*p* = 0.0001), and STAT3 (*p* < 0.00001); markers also known to be over-expressed in ER-positive breast cancer and associated with favourable outcome.^21,43^ By contrast, negative associations were observed with the proliferation marker Ki67 (*p* = 0.014), epithelial–mesenchymal transition markers such as N-cadherin (*p* < 0.00001) and phosphatidylinositol-4,5-Bisphosphate 3-Kinase Catalytic Subunit Alpha (PIK3CA; *p* = 0.012). In addition, the mediator subunit MED7 was positively associated (*p* < 0.00001) with RXRG (Table 2; both whole and ER-positive cohort). In ER-negative tumours, only MED7 (*p* < 0.00001), BEX1 (*p* = 0.032) and N-cadherin (*p* = 0.034) showed significant association with RXRG (Table 2).

**Table 2:** Associations between RXRG expression and other biomarkers in the whole series, in ER-positive and ER-negative breast cancer series

Parameters	RXRG expression whole cohort			RXRG expression ER-positive cohort			RXRG expression ER-negative cohort		
	Negative/low expression *N* (%)	High expression *N* (%)	*p*-value (*χ*^2^)	Negative/low expression *N* (%)	High expression *N* (%)	*p*-value (*χ*^2^)	Negative/low expression *N* (%)	High expression *N* (%)	*p-*value (*χ*^2^)
Oestrogen (ER) status									
Negative	138 (63.0)	81 (37.0)	**<0.0001** (20.142)						
Positive	319 (48.7)	380 (54.4)							
Progesterone (PR) status									
Negative	201 (56.3)	156 (43.7)	**0.018** (7.726)	67 (45.0)	82 (55.0)	0.780 (0.137)	134 (65.0)	73.0 (35.0)	1.00 (0.543)
Positive	247 (46.8)	281 (53.2)		246 (46.7)	281 (53.3)		1 (100.0)	0 (0.00)	
Human epidermal growth factor receptor 2 (HER2)									
Negative	371 (48.4)	395 (51.6)	0.057 (3.612)	269 (44.2)	339 (55.8)	0.102 5.750)	102 (66.0)	53 (34.0)	0.016 (1.928)
Positive	72 (57.6)	53 (42.4)		41 (59.4)	28 (40.6)		31 (55.0)	25 (45.0)	
Forkhead box protein A1 (FOXA1)									
Negative	235 (65.1)	126 (34.9)	<**0.00001** (33.053)	133 (61.0)	85 (39.0)	**<0.0001** (19.026)	102 (71.0)	41 (29.0)	0.194 (2.220)
Positive	103 (41.5)	145 (58.5)		92 (40.4)	136 (59.6)		11 (55.0)	9 (45.0)	
GATA-binding protein 3 (GATA3)									
Negative	266 (62.3)	161 (37.7)	<**0.00001** (36.024)	169 (58.9)	118 (41.1)	**0.0001** (23.251	97 (69.3)	43 (30.7)	0.312 (2.220)
Positive	43 (32.6)	89 (67.4)		43 (3.3)	86 (66.7)		0 (0.00)	1(100)	
Brain-expressed X-linked protein 1(BEX1)									
Negative	149 (70.0)	64 (30.0)	<**0.00001** (31.812)	99 (67.3)	48 (32.7)	**<0.00001** (24.131)	50 (77.0)	15 (23.0)	**0.032 (**5.610)
Positive	184 (46.1)	215 (53.9)		133 (42.8)	178 (57.2)		51 (59.0)	36 (41.0)	
Cluster of differentiation 71 (CD71)									
Negative	139 (50.2)	138 (49.8)	**0.049** (4.891)	115 (47.1)	129 (52.9)	0.496 (2.396)	25 (71.0)	10 (29.0)	0.838 (0.114)
Positive	218 (58.9)	152 (41.1)		130 (54.2)	110 (45.8)		89 (69.0)	41 (32.0)	
Ki67									
Negative	120 (41.0)	173 (59.0)	**0.0004** (15.903)	104 (39.4)	160 (60.6)	**0.014** (9.660)	15 (56.0)	12 (44.0)	0.678 (0.590)
Positive	240 (56.1)	188 (43.9)		150 (25.6)	135 (47.4)		90 (63.0)	52 (37.0)	
Cytokeartin5/6 (CK5/6)									
Negative	298 (49.1)	309 (50.9)	**0.020** (7.883)	242 (46.7)	276 (53.3)	1.63 (0.157)	56 (63.0)	32 (37.0)	0.623 (0.402)
Positive	70 (63.6)	40 (36.4)		8 (42.1)	11 (57.9)		62 (68.0)	29 (32.0)	
Phosphatidylinositol-4,5-bisphosphate 3-kinase, catalytic subunit alpha (PIK3CA)									
Negative	71 (40.1)	106 (59.9)	**0.0004** (15.545)	60 (38.7)	95 (61.3)	**0.012** (10.045)	11 (55.0)	9 (45.0)	0.458 (0.832)
Positive	307 (57.2)	230 (42.8)		205 (53.8)	176 (46.2)		102 (65.0)	54 (35.0)	
N-cadherin									
Negative	66 (34.2)	127 (65.8)	<**0.00001** (32.387)	53 (32.3)	111 (67.7)	**<0.00001** (20.774)	13 (46.0)	15 (54.0)	**0.034** (6.434)
Positive	286 (58.4)	204 (41.6)		194 (53.7)	167 (46.3)		92 (71.0)	37 (29.0)	
Signal transducer and activator of transcription 3 (STAT3)									
Negative	283 (59.7)	191 (40.3)	<**0.00001** (35.589)	197 (57.3)	147 (42.7)	<**0.00001** (28.678)	86 (66.0)	44 (34.0)	0.210 (1.734)
Positive	61 (34.3)	117 (65.7)		45 (30.8)	101 (69.2)		16 (53.0)	14 (47.0)	
Mediator of RNA polymerase II transcription subunit 7 **(MED7)**									
Negative	275 (67.7)	131 (32.3)	<**0.00001** (105.75)	117 (63.4)	102 (36.6)	<**0.00001** (68.053)	97 (79.0)	26 (21.0)	**<0.00001** (32.610)
Positive	105 (30.2)	243 (69.8)		81 (28.7)	201 (71.3)		24 (37.0)	41 (63.0)	

Significant *p-*values are highlighted in bold

Positive associations were observed between the nuclear expression of RXRG and the expression of nuclear receptors, including PPARγ, PPARβ, AR, RARα, glucocorticoid receptor and liver receptor homologue-1 (*p* for all <0.001) (Table 3; in the whole cohort, ER-positive and ER-negative cohort). Moreover, using the continuous *H*-score to assess the association between RXRG expression and the clinicopathological parameters, as well as other breast cancer-related biomarkers revealed similar significant association to those obtained with the categorised RXRG (Supplementary Table 1).

**Table 3:** Associations between RXRG expression and other nuclear receptors in the whole series, ER-positive and ER-negative breast cancer series

Parameters	Whole cohort			ER-positive cohort			ER-negative cohort		
	Negative/ low expression *N* (%)	High expression *N* (%)	*p*-value (*χ*^2^)	Negative/ low expression *N* (%)	High expression *N* (%)	*p-*value (*χ*^2^)	Negative/ low expression *N* (%)	High expression *N* (%)	*p-*value (*χ*^2^)
Androgen receptor (AR)									
Negative	253 (70.7)	105 (29.3)	**<0.0001** (105.72)	156 (70.0)	69 (30.0)	**<0.0001** (77.25)	97 (74.0)	34 (26.0)	**0.0003**(15.66)
Positive	103 (31.4)	225 (68.6)		88 (30.3)	202 (69.7)		14 (39.0)	22 (61.0)	
Glucocorticoid receptor (GR)									
Negative	184 (71.0)	75 (29.0)	**<0.0001** (67.10)	108 (66.0)	57 (34.0)	**<0.0001** (36.88)	76 (82.0)	17 (18.0)	**0.00001**(22.52)
Positive	129 (37.4)	216 (62.6)		100 (36.0)	180 (64.0)		28 (45.0)	34 (55.0)	
Liver receptor homologue-1(LRH-1)									
Negative	220 (65.5)	116 (34.5)	**<0.0001** (45.94)	142 (63.0)	85 (37.0)	**<0.0001** (34.53)	77 (73.0)	29 (27.0)	**0.039** (5.13)
Positive	135 (39.5)	207 (60.5)		103 (36.0)	180 (64.0)		32 (55.2)	26 (44.8)	
Peroxisome proliferator-activated receptor beta (PPARβ)									
Negative	227 (67.0)	112 (33.0)	<**0.00001** (59.84)	142 (64.0)	80 (36.0)	**<0.0001** (40.83)	85 (74.0)	30 (26.0)	**0.004** (10.556)
Positive	94 (35.3)	172 (64.7)		78 (34.0)	152 (66.0)		15 (44.0)	19 (56.0)	
Peroxisome proliferator-activated receptor gamma (PPARγ)									
Negative	267 (69.0)	120 (31.0)	<**0.00001** (107.54)	175 (67.0)	86 (33.0)	**<0.0001** (77.30)	92 (74.0)	33 (26.0)	**0.00001** (24.55)
Positive	51 (25.0)	15 7 (75.0)		437(25.0)	141 (75.0)		3 (15.8)	16 (84.2)	
Retinoid A receptor alpha (RARa)									
Negative	238 (68.0)	114 (32.0)	<**0.00001** (72.29)	193 (50.0)	194 (50.0)	**<0.00001** (24.13)	85 (80.0)	21 (20.0)	**<0.00001** (22.46)
Positive	117 (35.0)	216 (65.0)		52 (37.0)	88 (63.0)		26 (44.0)	33 (56.0)	
Retinoic acid-related orphan receptor gamma (RORγ)									
Negative	294 (55.0)	244 (45.0)	**0.002** (13.58)	115 (47.1)	129 (52.9)	**0.033** (6.69)	100 (68.0)	47 (32.0)	*p* = 0.22 (2.979)
Positive	60 (38.0)	98 (62.0)		130 (54.2)	110 (45.8)		8 (47.1)	9 (52.9)	
Vitamin D receptor (VDR)									
Negative	216 (59.0)	153 (41.0)	**0.004** (12.85)	133 (52.0)	121 (48.0)	0.090 (3.16)	82 (72.6)	31 (27.4)	**0.014** (10.309)
Positive	145 (45.0)	178 (55.0)		119 (45.0)	148 (55.0)		26 (47.3)	29 (52.7)	
Photoreceptor cell-specific nuclear receptor (PNR)									
Negative	206 (56.0)	161 (44.0)	**0.030** (5.09)	148 (52.0)	138 (48.0)	**0.042** (4.80)	57 (73.0)	21 (27.0)	0.22 (2.334**)**
Positive	162 (48.0)	178 (52.0)		103 (43.0)	141 (57.0)		59 (62.0)	36 (38.0)	

Significant *p*-values are highlighted in bold

### Association between RXRG protein expression and patients’ outcome

**Fig. 2:**
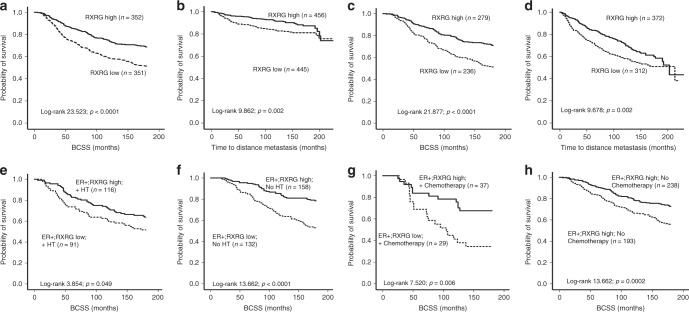
Kaplan–Meier plot for the association of RXRG nuclear expression. Whole series: **a** Breast cancer-specific survival, **b** distant metastasis-free survival. In ER-positive tumours. **c** Breast cancer-specific survival, **d** distant metastasis-free survival. Kaplan–Meier analysis of breast cancer-specific survival showing the impact of treatment on RXRG nuclear protein expression in ER-positive cohort; **e** in patients who did receive hormone therapy **f** in patients that did not receive hormone therapy **g** in patients who did receive chemotherapy and **h** in patients who did not receive chemotherapy with significance determined using the log-rank test

High expression of RXRG was associated with longer breast cancer-specific survival (*p* < 0.0001; Fig. 2a). Regarding distant metastasis, high RXRG expression was associated with a lower probability of distant metastasis (*p* = 0.002; Fig. 2b). Cox proportional multivariate analysis showed that RXRG expression was an independent indicator of both longer breast cancer-specific survival and distant metastasis-free interval in the whole cohort (HR = 0.6; 95% CI = 0.4–0.8; *p* = 0.04 and HR = 0.7; 95% CI = 0.6–0.9; *p* = 0.025, respectively) independent of the standard prognostic parameters of breast cancer, including tumour size, histological grade, nodal stage, ER status and proliferative fraction as assessed by Ki67. Comparable results were obtained when we included the ER level of expression as a continuous variable to the multivariate analysis of the ER-positive cohort (Table 4).

**Table 4:** Univariate and multivariate analysis of RXRG expression compared with tumour stage, grade, size, Ki67and ER status for breast cancer-specific survival and distant metastasis-free survival

Variable	Breast cancer-specific survival						Distant metastasis-free interval					
	Univariate			Multivariate			Univariate			Multivariate		
	HR	95% CI	*p-*value	HR	95% CI	*p-*value	HR	95% CI	*p*-value	HR	95% CI	*p-*value
Whole cohort												
Stage	2.1	1.9–2.4	**<0.0001**	2.2	1.7– 2.8	**<0.0001**	2.3	2.1–2.5	**<0.0001**	2.0	1.6–2.4	**<0.0001**
Grade	2.3	2.0–2.6	**<0.0001**	1.7	1.3– 2.5	**<0.0001**	1.7	1.6–2.0	**<0.0001**	1.3	1.1–1.6	**0.039**
Tumour size	2.1	1.8–2.5	**<0.0001**	1.6	1.1–2.2	**0.006**	1.9	1.6–2.2	**<0.0001**	1.4	1.1–1.9	**0.005**
ER^a^	0.9	0.9–1.1	**<0.0001**	1.1	0.9–1.2	0.558	0.9	0.8–1.1	**<0.0001**	1.6	1.1–2.3	**0.026**
Ki67	2.6	2.1–3.1	**<0.0001**	1.5	1.1–2.3	**0.027**	2.1	1.7–2.5	**<0.0001**	1.6	1.2–2.2	**0.004**
RXRG	0.6	0.4–0.7	**<0.0001**	0.6	0.4–0.8	**0.040**	0.8	0.6–0.9	**0.003**	0.7	0.6–0.9	**0.025**
ER+cohort												
Stage	2.0	1.8–2.4	**<0.0001**	2.1	1.6–2.7	**<0.0001**	2.2	1.9–2.4	**<0.0001**	2.0	1.6–2.4	**<0.0001**
Grade	2.4	2.1–2.8	**<0.0001**	1.6	1.2–2.3	**0.004**	1.9	1.6–2.1	**<0.0001**	1.3	0.9–1.7	0.084
Tumour size	2.3	1.9–2.9	**<0.0001**	1.6	1.1–2.4	**0.025**	2.2	1.8–2.6	**<0.0001**	1.5	1.1–2.1	**0.024**
ER^a^	0.9	0.9–1.0	0.101	0.9	0.8–1.1	0.428	1.0	0.9–1.2	**0.002**	0.9	0.8–1.1	0.456
Ki67	2.9	2.3–3.7	**<0.0001**	1.8	1.2–2.9	**0.005**	2.4	1.9–3.0	**<0.0001**	1.8	1.2–2.6	**0.002**
RXRG	0.5	0.4–0.7	**<0.0001**	0.5	0.4–0.7	**0.004**	0.7	0.5–0.9	**0.002**	0.7	0.5–0.9	**0.036**

Significant *p-*values highlighted in bold

^a^ER used as a continuous variable (percentage of positive tumour cells)

Similarly, in ER-positive tumours, high RXRG levels were predictive of longer breast cancer-specific survival (*p* < 0.0001; Fig. 2c) and longer distant metastasis-free interval (*p* = 0.002; Fig. 2d). The Cox regression model demonstrated that RXRG was an independent predictor of both breast cancer-specific survival and longer distant metastasis-free interval (HR = 0.5; 95% CI = 0.4–0.7; *p* = 0.004 and HR = 0.7; 95% CI = 0.5–0.9; *p* = 0.036, respectively, Table 4). In triple-negative breast cancer and HER2+ phenotypes, RXRG expression was neither associated with breast cancer-specific survival nor with distant metastasis-free interval.

RXRG positivity was associated with a significant survival advantage in patients with ER-positive tumours irrespective of hormonal therapy (*p* = 0.049 and *p* < 0.0001, respectively, Fig. 2e, f). Similarly, in ER-positive patients who either received or did not receive adjuvant chemotherapy, the prognostic advantage of positive RXRG expression was maintained (*p* = 0.006 and *p* = 0.002, respectively) (Fig. 2g, h). Supporting this, evaluation of the interaction between RXRG and ER level of expression (RXRG*ER) using the Cox regression model showed significant association with longer breast cancer-specific survival and distant metastasis-free interval (both *p* = 0.001).

There was a trend towards a positive linear correlation between *RXRG* mRNA and protein expression in the subset of Nottingham cases within the METABRIC study (*n* = 150), that has data on both mRNA and protein expression, however, the association did not reach statistical significance (*r* = 0.20, *p* = 0.077).

### Genomic study and pathway analysis

We next identified differential gene expression between patients with low versus high RXRG mRNA expression in the Nottingham primary operable breast cancer series, which were included in the METABRIC^34^ study (*n* = 150). This analysis identified 1048 significant differentially expressed genes (*p* < 0.05), comprises of 554 over-expressed and 494 downregulated genes, associated with reduced RXRG expression. Analysis of the differential gene expression list identified over-represented pathways, including dysregulation of genes regulating ER signalling pathway (Supplementary Table 2; *p* = 0.0053; *FOS* and *AP-1* transcription factor subunit). Other relevant pathways involved in regulating RXRG protein expression included cAMP signalling pathway (*p* = 0.001; *ADORA1*), protein digestion and absorption pathway (*p* = 0.001; *COL4A2* and *SLC7A7* and the ABC transport pathway (*p* = 0.002; *ABCB9* and *ABCD3*). Interaction with RXRG and other nuclear receptors in steroid signalling pathways are summarised in Supplementary Fig. 2.

### *RXRG* genomic profiling

Expression analysis for *RXRG* mRNA using Breast Cancer Gene-Expression Miner v4.0 showed that high *RXRG* expression was associated with older age at diagnosis (*n* = 3600; Supplementary Fig. 3a; *p* = 0.0082), lower histological tumour grade (*n* = 3518; *p* = 0.0024; Supplementary Fig. 3b), ER-positive status (*n* = 5558; Supplementary Fig. 3c; *p* = 0.029). Among PAM50 subtypes, *RXRG* mRNA was associated with luminal subtypes (*n* = 5607; *p* = 0.0024; Supplementary Fig. 3d) and non-triple-negative status (*n* = 1275; *p* = 0.014; Supplementary Fig. 3e). Targeted prognostic analyses for *RXRG* with nodal status and positive ER status patients (*n* = 33 data sets, 3941 patients) indicated that high gene expression correlated with adverse event-free survival (HR = 0.88; 95% CI = 0.79–0.98; *p* = 0.025; Supplementary Fig. 3f). Consistent with this, Kaplan–Meier analysis^38^ indicates high *RXRG* expression showed significant survival advantage irrespective of systemic treatment in (*n* = 3951; *p* < 0.0001; Supplementary Fig. 3g). To confirm this, we examined the TCGA-BRCA^44,45^ data set and found high *RXRG* mRNA expression was associated with longer disease-free intervals, post-menopausal status, and differential ER, PR and HER2 expression (Supplementary Fig. 4a–f).

## Discussion

Understanding the mechanisms by which RXRs exert their effects in breast cancer remains incomplete.^12^ To our knowledge, this is the first study to define the prognostic role RXRG in breast cancer using a large clinical data set with long-term follow up. Results from the current study provide evidence that high expression of RXRG protein was significantly associated good long-term clinical outcome. Our study shows that high-nuclear RXRG was associated with ER-positive tumours, and is consistent with previous reports, which shows it confers a better prognostic impact.^46^ Indeed, the positive correlation between RXRG and ER expression, and association of higher RXRG with improved patient outcome independent of ER expression, suggest that RXRG could be a potential surrogate marker for ER expression in our cohort. Moreover, RXRG expression is significantly higher in breast cancer histologic subtypes with better prognosis such as invasive lobular carcinoma,^46,47^ in contrast to ductal or medullary-like tumours, which typically are associated with poorer outcomes.

In this study, ER-positive breast cancer showed the highest expression of RXRG compared to HER2+ and triple-negative breast cancer. Moreover, elevated expression of RXRG was associated with ER associated markers, such as GATA3,^48^ FOXA1,^49^ BEX,^30^ STAT3^43^ and MED7.^33^ As noted earlier, RXRs and RARs form heterodimeric complexes, which bind DNA at specific retinoid responsive elements and regulate the various transcriptional processes.^12^ In breast cancer, functional interactions between retinoic acid and oestrogen signalling are complex and well documented.^2,19,20^

In this study pathway, analyses were conducted to explore the differentially enriched pathways associated with increased expression levels of RXRG protein. Results on pathway analysis confirmed our IHC findings reinforcing the importance of RXRG expression and ER status, where it revealed a positive association between high RXRG expression and ER positivity, and on patients’ survival. Our results indicated that the ER enriched pathway was the top master regulator of RXRG. Thus, we exposed a positive correlation between the genes regulating the ER pathway and RXRG protein expression, suggesting that suppressed expression of those indicators may inhibit signalling via the ER pathway and consequently affecting RXRG expression. For instance, dimerised ER directly binds to DNA sequences called oestrogen response elements (EREs) in relevant activated genes and activate gene transcription. However, ER is also known to use non-classical pathways via Activator protein 1 (AP-1) or via Specificity protein 1 (Sp-1).^50^ In ER-positive, breast cancer cell lines, ER enhanced *ADORA1* mRNA and protein levels. Inhibition of *ADORA1* reduced the binding activity of ER to its target gene indicating its role for the transcriptional activity of ER on oestrogen stimulation.^51^ By decreasing *COL4A2* mRNA levels through miR-29b may be contribute to the tumorigenicity in ER-positive BC cells.^52^ The aforementioned studies have revealed the potential role of these biomarkers in ER-related pathways and may affect RXRG expression. However, it is important to note that the role of RXRG within ER-related pathways may be quite complex, depending on the specific interacting partners. For example, in this study, RXRG expression was negatively associated with PIK3CA. *PIK3CA* mutations are strongly associated with ER-positive tumours with better prognostic characteristics.^53^ Thus, its inverse relationship to PIK3CA warrants further investigation in the context of ER-associated pathways. Interestingly, in the MNU-induced rat mammary tumour models, the RXR-selective retinoid bexarotene (Targretin), suppressed ER-positive tumour development with minimal toxicity.^54^

In this study, the negative correlation with N-cadherin, CK5/6 and Ki67 indicates that RXRG expression is not associated with aggressive breast cancers. Elevated N-cadherin expression is associated with epithelial–mesenchymal transition (EMT) and tumour aggressiveness.^55^ In thyroid carcinoma, administration of ligands selective for RXRG resulted in a 30% reduction in cell proliferation,^56^ which is in agreement with low proliferation index and high RXRG expression. High molecular weight cytokeratin are strongly associated with high histological grade, and worse patient outcome^31^ and their negative association with RXRG further reinforces its role as a good prognostic indicator.

Nuclear RXRG expression displayed strong positive associations with other nuclear receptors. Studies have shown that RXRs form heterodimers with many nuclear receptors, including RARs, VDRs, PPARs, liver-x receptor (LXRs) and farnesoid X receptors (FXRs),^57^ suggesting that the positive correlations in our study could be due to heterodimer formation with one or more of these nuclear receptors. For instance, in breast cancer cells treated with ligands specific for PPARγ and RXR/RAR, troglitazone and 9-*cis*-retinoic acid, respectively, a reduction in proliferation was observed,^58^ and low doses of PPARγ and RXR ligands also promoted apoptosis.^59^ This suggests that RXRs have an anti-tumorigenic role, potentially through heterodimer formation with PPARγ. Treatment of thyroid cancer cells containing both RXRG and PPARγ with their ligands resulted in a synergistic increase in apoptotic activity.^56^ This suggests that RXRγ-PPARγ heterodimer may be present, and that the activation of this heterodimer leads to a synergistic increase in apoptosis. For this reason, we propose that increased expression of RXRG could potentiate heterodimer formation and activation of other nuclear receptors (e.g., VDR, RAR and PPARγ) thereby enhancing their anti-tumorigenic functions.

Regarding the association with patient outcome, high-nuclear RXRG expression was associated with improved breast cancer-specific survival and a longer time to distant metastasis in the whole series and in ER-positive breast cancer. However, in other breast cancer subtypes RXRG did not show any association with patient outcome. This might be due to the smaller sample size of ER-negative, HER2+ and triple-negative breast tumours in this cohort. Further investigation of larger cohorts of ER-negative, HER2+, and triple-negative breast tumours is therefore warranted. Our findings are consistent with previous reports in breast and renal cancer.^60,61^ In our study, these outcome associations were independent of other well-established prognostic variables. Interestingly, increased RXRG expression showed improved outcome regardless of adjuvant hormonal therapy or chemotherapy status. Hence, in chemotherapy-intolerant patients, therapeutic manipulation of RXRG on its own, or in combination with other therapies, may be helpful in improving the existing treatment regimen, particularly as next-generation RXR subtype-selective rexinoids enter clinical testing and use. Furthermore, assessment of *RXRG* mRNA levels using bc-GenExMiner and TCGA demonstrated that high *RXRG* mRNA expression is significantly associated with better tumour characteristics and longer event-free survival of breast cancer patients, which corroborates with RXRG protein expression. *RARA* mRNA expression levels in breast cancer patients treated with hormonal therapy predicted positive outcome,^19^ which is in agreement with our findings.

In summary, high RXRG expression in breast cancer is associated with favourable prognostic parameters and is an independent prognostic factor with prolonged patient survival. The interaction between RXRG, ER, and other nuclear receptors may explain the prognostic effect of RXRG in breast cancer. There is evidence that rexinoids are more effective anti-cancer agents than retinoids in preclinical models and show minimal toxicity.^62^ Therefore, further studies to validate the potential of RXRG as a therapeutic target in breast cancer are therefore warranted.

## Supplementary information

Supplementary Tables and Figures

## Data Availability

The authors confirm the data that has been used in this work is available on reasonable request.
