# Supplementary material for: Retinoid X receptor gamma (RXRG) is an independent prognostic biomarker in ER-positive invasive breast cancer
Source: Br J Cancer. 2019 Sep 27;121(9):776–85. doi: 10.1038/s41416-019-0589-0 (PMC6889395; doi:10.1038/s41416-019-0589-0)
Supplement: Supplementary file 1 — Supplementary Tables and Figures [file 41416_2019_589_MOESM1_ESM.docx]

**Supplementary Tables**

**Supplementary Table 1:** Association between RXRG expression and clinico pathological parameters and other related biomarker using the continuous H-score.

| **Parameters** | **RXRG expression Whole cohort** | | |
| --- | --- | --- | --- |
|  | **N (%)** | **Mean Rank** | **P value** |
| **Oestrogen (ER) status** | | | |
| Negative | 219 (23.0) | 384.35 | **<0.0001** |
| Positive | 699 (77.0) | 483.05 |  |
| **Progesterone (PR) status** | | | |
| Negative | 357 (40.0) | 413.97 | **0.005** |
| Positive | 528 (60.0) | 462.63 |  |
| **Human epidermal growth factor receptor 2 (HER2)** | | | |
| Negative | 766 (86.0) | 453.45 | **0.032** |
| Positive | 125 (14.0) | 400.36 |  |
| **Histological type** | | | |
| Ductal | 756 (82.8) | 442.64 | **<0.0001** |
| Lobular | 99 (11.3) | 551.63 |  |
| Medullary-like | 21 (3.0) | 411.62 |  |
| Special type | 36 (2.9) | 620.19 |  |
| **Nottingham Prognostic Index** | | | |
| Good Prognostic Group | 268 (29.3) | 517.96 | **<0.0001** |
| Moderate Prognostic Group | 495 (54.0) | 439.04 |  |
| Poor Prognostic Group | 153 (16.7) | 147.29 |  |
| **Tumour size** | |  | |
| ˂ 2.0cm | 425 (46.3) | 497.84 | **<0.0001** |
| ≥ 2.0cm | 491 (53.7) | 424.45 |  |
| **IHC subtype** | | | |
| ER+/HER2- Low Proliferation | 214 (27.2) | 461.20 | **<0.0001** |
| ER+/HER2- High Proliferation | 292 (37.4) | 393.11 |  |
| Triple Negative | 150 (19.2) | 311.50 |  |
| HER2+ | 124 (15.8) | 357.90 |  |
| **Forkhead box protein A1 (FOXA1)** | | | |
| Negative | 361(59.0) | 271.02 | <**0.0001** |
| Positive | 248 (41.0) | 354.46 |  |
| **GATA binding protein 3 (GATA3)** | | | |
| Negative | 427 (76.0) | 254.76 | <**0.0001** |
| Positive | 132 (23.0) | 361.64 |  |
| **Brain-expressed X-linked protein 1(BEX1)** | | | |
| Negative | 213 (35.0) | 247.92 | <**0.0001** |
| Positive | 399 (65.0) | 337.77 |  |
| **Cluster of Differentiation 71 (CD71)** | | | |
| Negative | 279 (43.0) | 345.06 | **0.025** |
| Positive | 372 (57.0) | 311.70 |  |
| **Ki67** | | | |
| Negative | 293 (41.0) | 400.88 | **<0.0001** |
| Positive | 428 (59.0) | 333.70 |  |
| **Cytokeartin5/6 (CK5/6)** | | | |
| Negative | 607 (85.0) | 368.71 | **0.003** |
| Positive | 110 (15.0) | 305.41 |  |
| **Phosphatidylinositol-4,5-bisphosphate 3-kinase, catalytic subunit alpha (PIK3CA)** | | | |
| Negative | 177 (25.0) | 399.13 | **0.002** |
| Positive | 537 (75.0) | 343.78 |  |
| **N cadherin** | | | |
| Negative | 193 (28.0) | 418.08 | **0.003** |
| Positive | 490 (72.0) | 312.03 |  |
| **Signal transducer and activator of transcription 3 (STAT3)** | | | |
| Negative | 474 (73.0) | 292.60 | <**0.0001** |
| Positive | 178 (27.0) | 416.77 |  |
| **Mediator of RNA polymerase II transcription subunit 7 (MED7)** | | | |
| Negative | 406 (54.0) | 287.78 | <**0.0001** |
| Positive | 348 (46.0) | 482.18 |  |

Significant p values are highlighted in bold

**Supplementary Table 2**: Pathway analysis RXRG expression.

| **ID** | **Master regulator** | **Genes** | **Enrichment score** | **Normalised Enrichment score** | **Leading Edge Genes** | **Gene Name** | **Score** | **FDR from GSEA** | **P-value (GSEA)** |
| --- | --- | --- | --- | --- | --- | --- | --- | --- | --- |
| Hsa04915 | Oestrogen signalling pathway | 7 | 0.910 | 1.24 | *FOS* | Fos proto-oncogene, AP-1 transcription factor subunit | 0.041 | 0.517 | **0.0053** |
| Hsa04024 | cAMP signalling pathway | 12 | 0.89 | 1.25 | *ADORA1* | adenosine A1 receptor | 0.041 | 0.0537 | **0.001** |
|  |  |  |  |  | *FOS* | Fos proto-oncogene, AP-1 transcription factor subunit | 0.035 |  |  |
| Hsa04974 | Protein Digestion and Absorption | 11 | 0.9 | 1.25 | *COL4A2* | Collagen type IV alpha 2 chain | 0.035 | 0.065 | **0.001** |
|  |  |  |  |  | *SLC7A7* | Solute carrier family 7 member 7 | 0.048 |  |  |
| Hsa02010 | ABC transporter | 6 | 0.94 | 1.28 | *ABCB9* | ATP binding cassette subfamily B member 9 | 0.047 | 0.091 | **0.002** |
|  |  |  |  |  | *ABCD3* | ATP binding cassette subfamily D member 3 | 0.037 |  |  |

**Supplementary Figures**


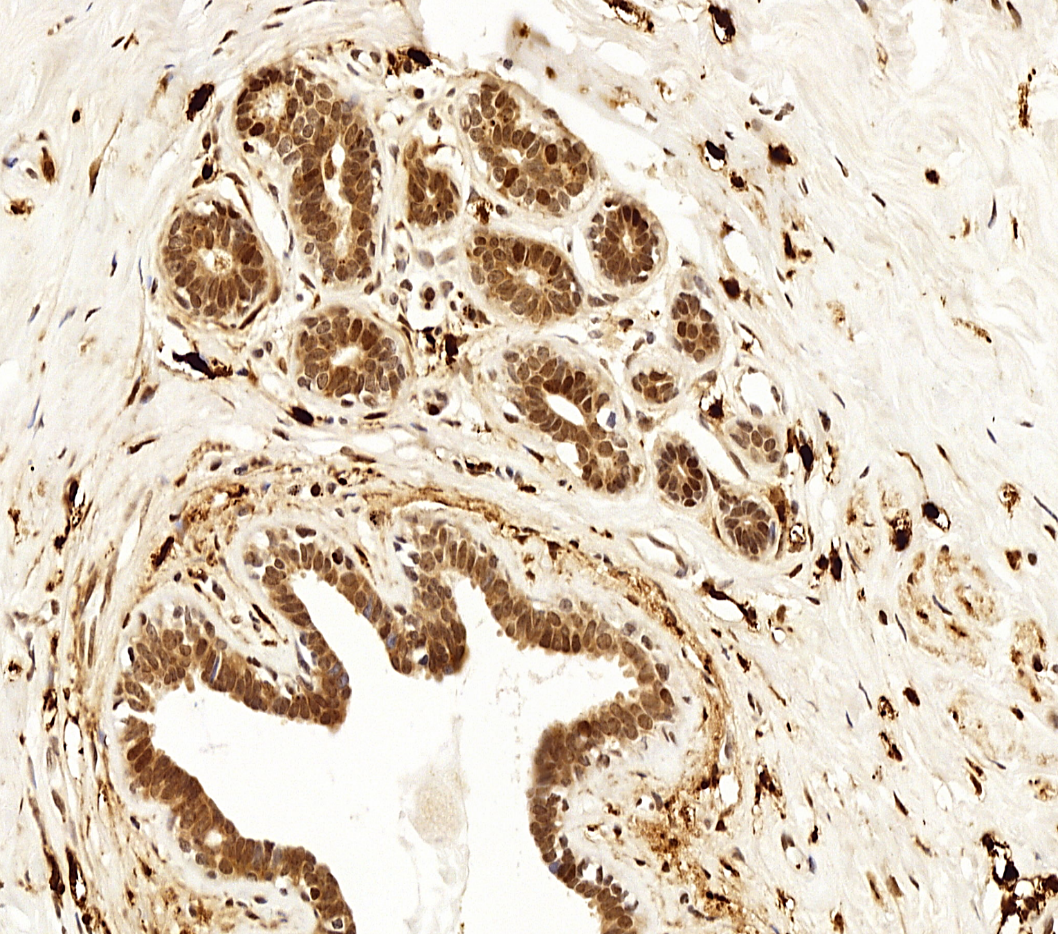

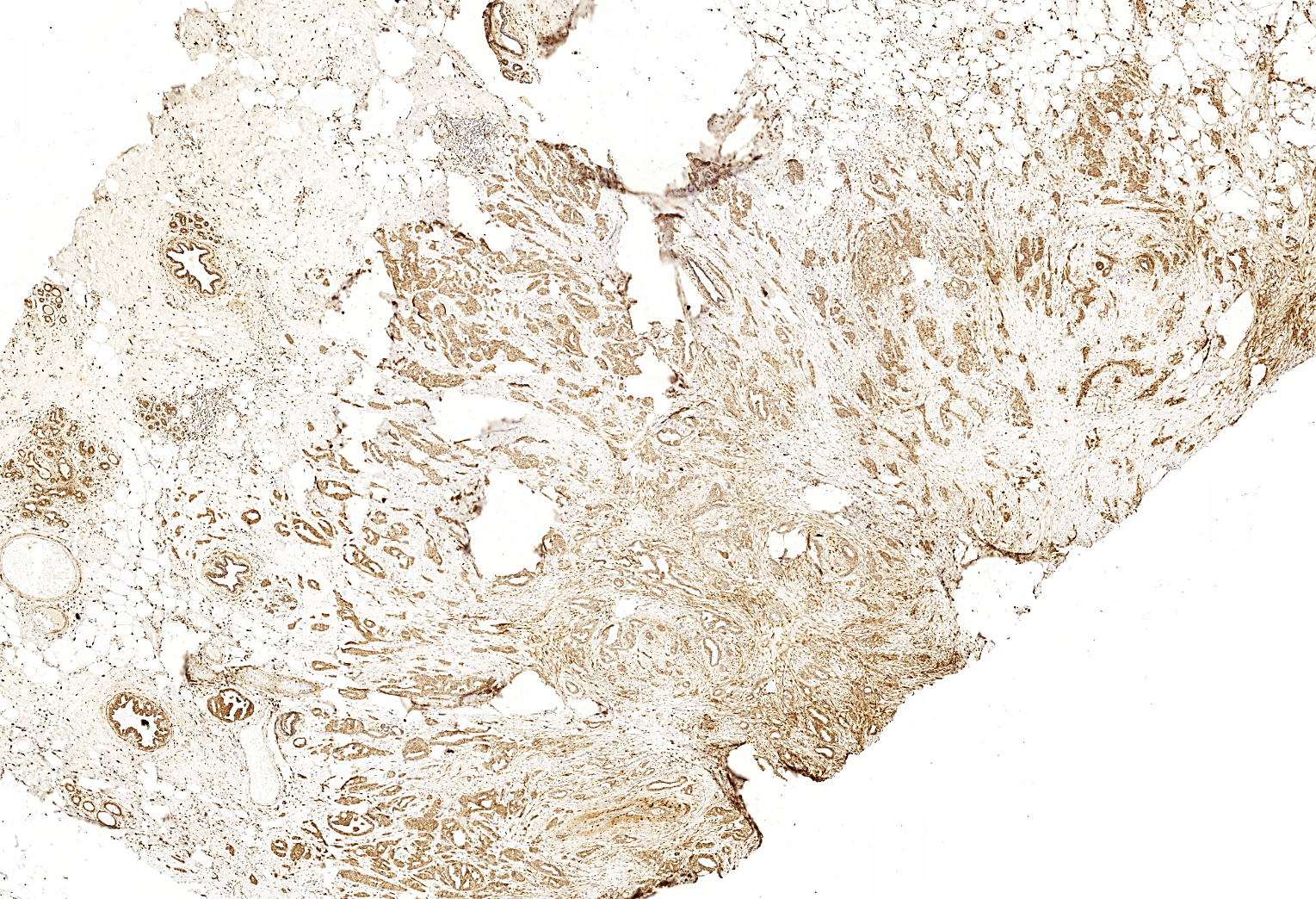

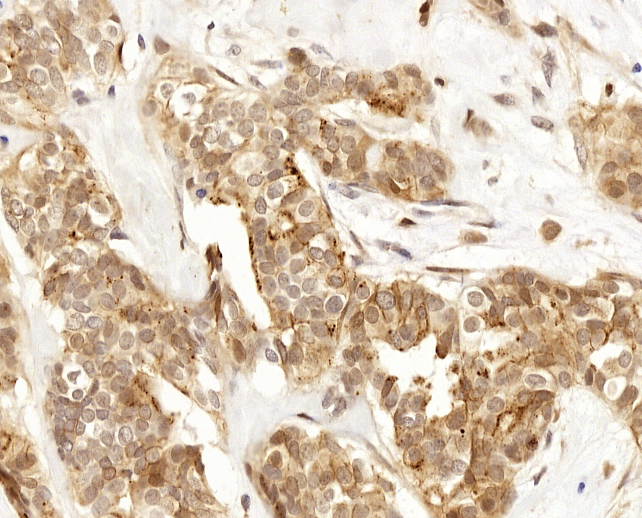


**Supplementary Figure 1: Morphological characteristics of RXRG immunohistochemistry in Full-face Breast cancer tissue.** (A) Showing the full-face breast cancer tissue with normal and invasive morphology. (B) Normal mammary gland showing strong RXRG nuclear immunoreactivity. (C) Weak RXRG nuclear immunoreactivity in invasive breast cancer.


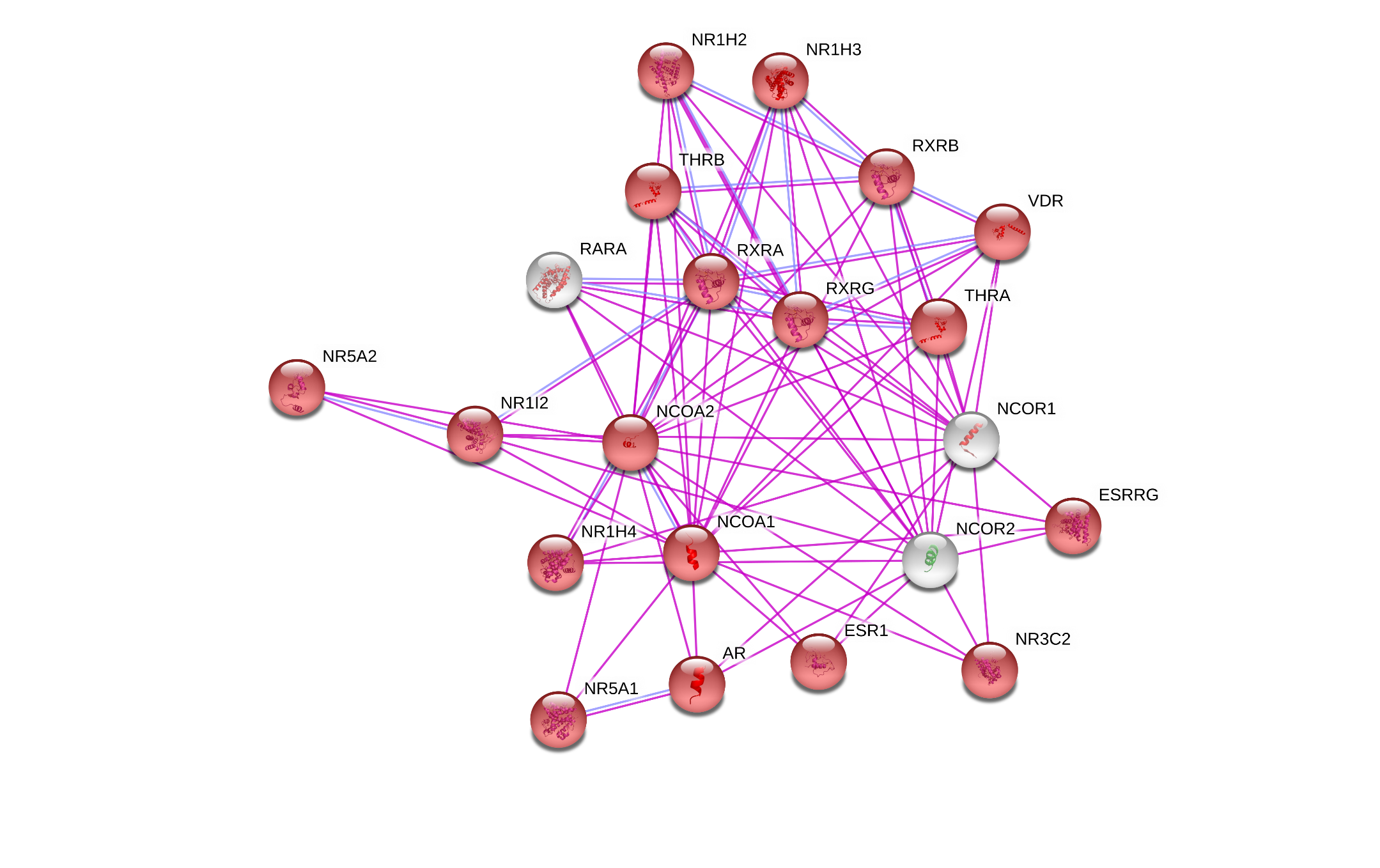


**Supplementary Figure 2.** RXRG protein interaction in steroid signaling pathway (using String 10). Protein interaction (PPI) enrichment (*p*=1x10^-16^).


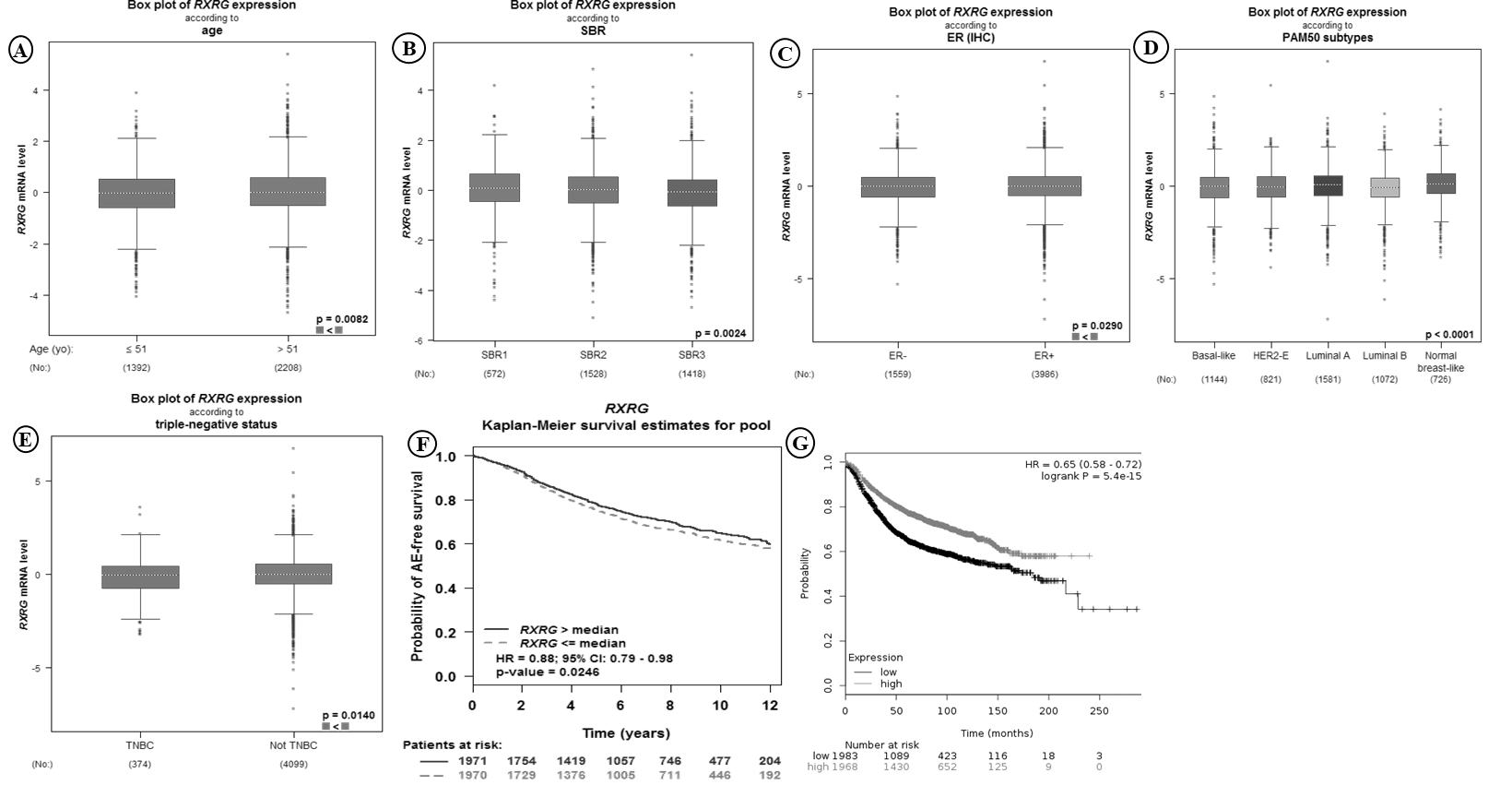


**Supplementary Figure 3**: **RXRG gene expression and its association, using Breast Cancer Gene-Expression Miner v4.0.** A) Age, B) Tumour grade C) ER status D) PAM50 subtype, E) Triple Negative status and F) Kaplan–Meier plots of RXRG gene expression. G) Patients with following systemic treatment (http://kmplot.com) either ET/chemotherapy showed high expression of RXRG associated with better patient survival.


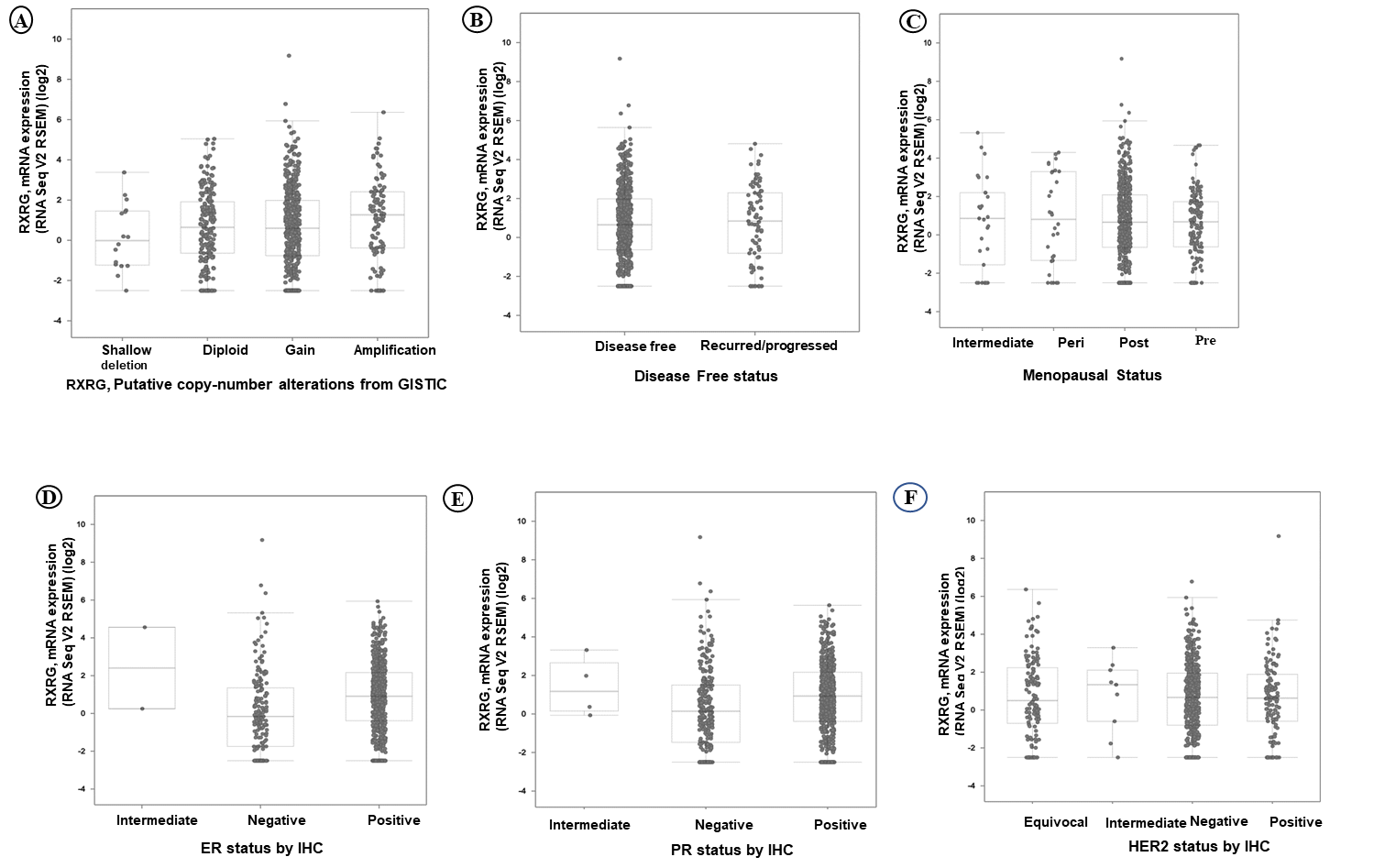


**Supplementary Figure 4. *RXRG gene* expression and its association, using TCGA data.**  A) Putative copy-number alterations B) Disease free status C) Menopausal status, D) ER status, E) PR status and F) HER2 status.
